# Facile Fabrication of Bio‐ and Dual‐Functional Poly(2‐oxazoline) Bottle‐Brush Brush Surfaces

**DOI:** 10.1002/chem.201905326

**Published:** 2020-02-12

**Authors:** Yunhao Du, Tao Zhang, Dan Gieseler, Maximilian Schneider, Daniel Hafner, Wenbo Sheng, Wei Li, Fred Lange, Erik Wegener, Ihsan Amin, Rainer Jordan

**Affiliations:** ^1^ Chair of Macromolecular Chemistry Faculty of Chemistry and Food Chemistry Technische Universität Dresden Mommsenstr. 4 01069 Dresden Germany; ^2^ Key Laboratory of Bio-based Polymeric Materials Technology and Application of Zhejiang Province Ningbo Institute of Material Technology and Engineering, Chinese Academy of Sciences Zhongguan West Road, 1219 315201 Ningbo China; ^3^ Van't Hoff Institute of Molecular Science, University of Amsterdam Science Park 904 1098 XH Amsterdam The Netherlands

**Keywords:** bio-functional surfaces, bottle-brush brushes, controlled radial polymerization, poly(2-oxazolines), polymer brush

## Abstract

Poly(2‐oxazoline)s (POx) bottle‐brush brushes have excellent biocompatible and lubricious properties, which are promising for the functionalization of surfaces for biomedical devices. Herein, a facile synthesis of POx is reported which is based bottle‐brush brushes (BBBs) on solid substrates. Initially, backbone brushes of poly(2‐isopropenyl‐2‐oxazoline) (PIPOx) were fabricated via surface initiated Cu^0^ plate‐mediated controlled radical polymerization (SI‐Cu^0^CRP). Poly(2‐methyl‐2‐oxazoline) (PMeOx) side chains were subsequently grafted from the PIPOx backbone via living cationic ring opening polymerization (LCROP), which result in ≈100 % increase in brush thickness (from 58 to 110 nm). The resultant BBBs shows tunable thickness up to 300 nm and high grafting density (*σ*) with 0.42 chains nm^−2^. The synthetic procedure of POx BBBs can be further simplified by using SI‐Cu^0^CRP with POx molecular brush as macromonomer (*M*_n_=536 g mol^−1^, PDI=1.10), which results in BBBs surface up to 60 nm with well‐defined molecular structure. Both procedures are significantly superior to the state‐of‐art approaches for the synthesis of POx BBBs, which are promising to design bio‐functional surfaces.

## Graphical abstract

**The facile fabrication** of POx BBBs with high layer thickness (up to ≈300 nm) and grafting density (*σ*=0.42 chains nm^−2^) via surface‐initiated Cu‐mediated controlled radical polymerization (SI‐Cu^0^CRP) is presented. The patterned and gradient POx BBB surfaces were fabricated in a straightforward manner. The dual functionalities of POx BBBs were demonstrated by fluorescent labelling.

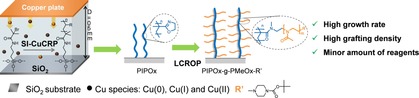

## Introduction

Polymer brushes with low protein adsorption and cell adhesion are receiving extensive attention since they are suitable for highly sensitive in vitro diagnostics and clean in vivo applications such as biomedical implants.^1^ So far, poly‐(ethylene glycol) (PEG) is one of the most widely utilized polymers for biomedical applications since it is bio‐inert and protein‐repellent.1a, 2 However, it has been reported that the PEGs can undergo oxidative degradation and cause antibodies against PEGs in vivo.^3^ Recently, poly(2‐oxazoline)s (POx)s have been established as a promising alternative material due to their good biocompatibility and durability.^4^ The reported POx brushes on surfaces that are composed of a linear back bone and densely grafted side chains are termed as bottle‐brush brushes (BBBs) due to their cylindrical appearances.4a A key advantage of the POx BBBs is dual‐functionality that enables to tune surface properties through the functionalization of the backbone as well as side chains.4a, 4d As such, POx BBB surfaces have been used in many biomedical applications such as recognition sites for cells, non‐fouling coatings against undesirable proteins and surface lubrications.4e, 4g, 5

**Figure 1:**
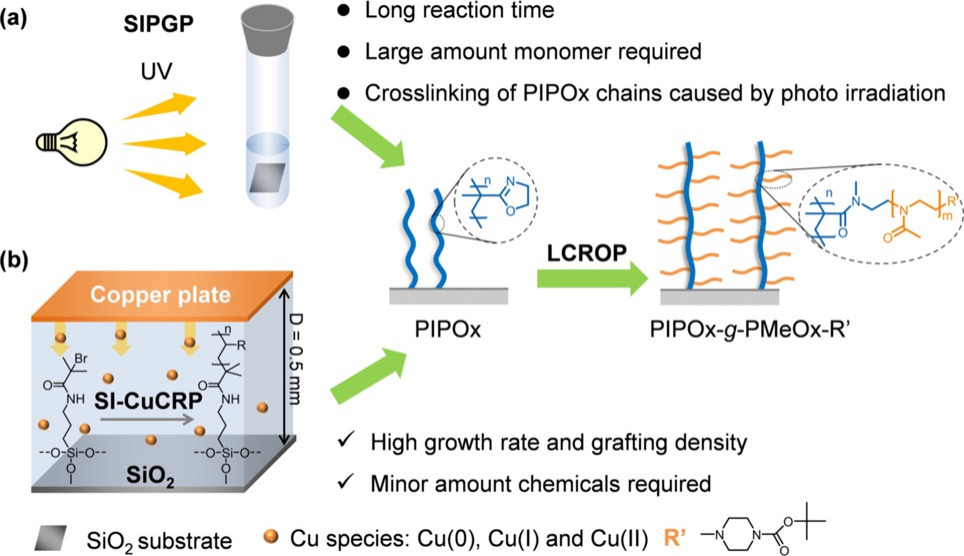
Schematic illustration of the synthesis of POx BBBs. (a) Conventional two‐step synthesis via SIPGP and SI‐Cu^0^CRP; (b) Two‐step synthesis via SI‐Cu^0^CRP and LCROP in this work. R=functional groups of different monomers. R′=terminating reagent for LCROP, *N*‐*tert*‐butoxycarbonyl piperazine (*N*‐Boc‐piperazine). D=distance between copper plate and initiating‐substrate.

Previously, we reported the synthesis of well‐defined POx BBBs via self‐initiated photografting and photopolymerization (SIPGP) and living cationic ring opening polymerization (LCROP).4a, 5b Firstly, poly(2‐isopropenyl‐2‐oxazoline) (PIPOx) backbone brushes from bulk IPOx monomers were synthesized via SIPGP by UV‐light irradiation (*λ*_max_=350 nm). The side chains were consecutively grafted from the PIPOx backbone brushes via LCROP using different 2‐alky‐oxazoline monomers (Figure 1 a).4a, 5b The SIPGP requires long reaction time (up to 40 h) and comparably large amount monomers (2 mL bulk monomer for each sample). Another challenge is that the already grafted POx brushes can be partially crosslinked by long‐term UV irradiation during SIPGP process.^6^ Therefore, a more controllable and reliable technique is highly needed.

The recently emerged SI‐Cu^0^CRP is a very effective and versatile technique to fabricate polymer brushes on planar substrates.^7^ The brush growth rate was found among the highest for surface‐initiated controlled radical polymerization reported to date.7h, 8 More importantly, the SI‐Cu^0^CRP is oxygen tolerant and requires very limited amount of monomers (μL), and therefore can be used to prepare polymer brushes with low cost.7a, 7j In addition, by simply variation of the distance (*D*) between the copper plate and the initiating surface, various structured polymer brushes can be prepared.7a, 7c, 7g, 7i

Here, we report the synthesis of well‐defined POx BBBs via a combination of SI‐Cu^0^CRP and LCROP. Initially, the PIPOx backbone brushes of up to ≈130 nm thickness were prepared by SI‐Cu^0^CRP at ambient conditions. Afterwards, the side group of PIPOx was extended via LCROP with 2‐methyl‐2‐oxazoline (MeOx) monomers and resulting in PIPOx‐*g*‐PMeOx BBBs with thickness up to ≈300 nm and high grafting density (*σ*=0.42 chains nm^−2^). The synthetic procedure towards POx BBBs can be further simplified by using SI‐Cu^0^CRP with POx molecular brush as macromonomer (*M*_n_=536 g mol^−1^, PDI=1.10), which results in BBBs surface in one grafting process with 60 nm thickness and well‐defined molecular structure. Both procedures improve the state of art approaches for the synthesis of well‐defined POxs BBBs, which are promising to design bio‐functional surfaces.

## Results and Discussion

The preparations of poly(2‐oxazoline) bottle‐brush brushes (POx BBBs) are outlined in Figure 1 b. The PIPOx backbone brush was synthesized via SI‐Cu^0^CRP, where a copper plate was sandwiched with an ATRP initiator‐modified SiO_2_ substrate at a typical distance of 0.5 mm and submerged in a reaction mixture containing 0.5 mL monomer (IPOx), 20 μL ligand (1,1,4,7,7‐pentamethyldiethylentriamin, PMDETA) and 1.5 mL solvent (water‐methanol, 2:1, v/v).7a, 7i Afterwards, the oxazoline groups of PIPOx backbones were converted under inert conditions (argon) using methyl triflate at −35 °C in dry acetonitrile (ACN) to the cationic macroinitiator poly(2‐isopropenyl‐2‐oxazolinium triflate). Then, side chain was prepared via LCROP at 80 °C for 4 h using 2‐methyl‐2‐oxazoline (MeOx) as monomer to form PIPOx‐*g*‐PMeOx BBBs. Comparing to classical SIPGP approach, the SI‐Cu^0^CRP is more efficient, controllable and requires only minor amount of chemicals (μL), since the reaction is confined between the copper plate and initiating‐substrate.7a

### Homogeneous POx BBB surfaces

**Figure 2:**
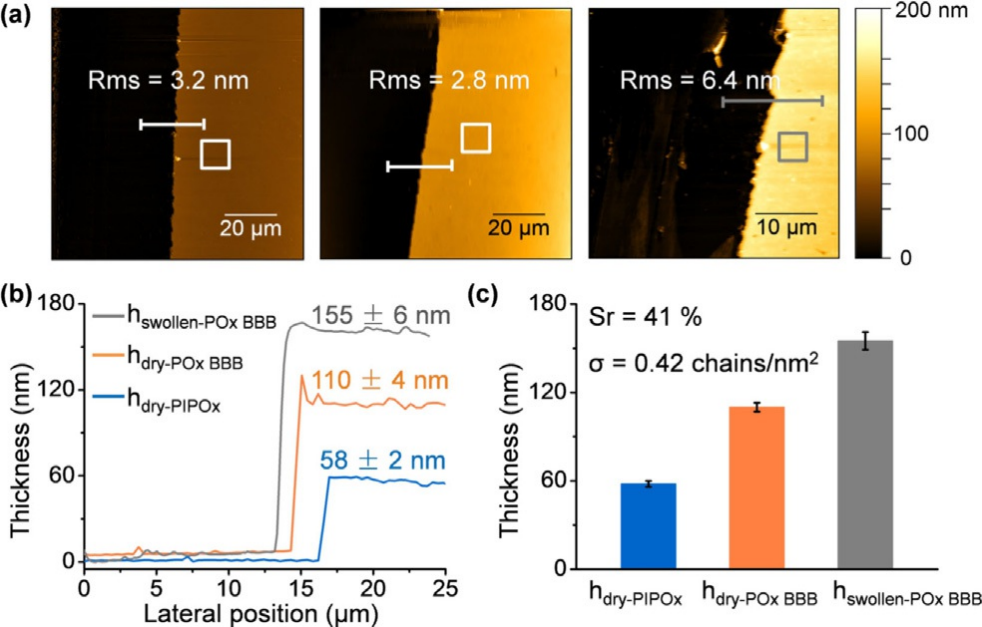
(a) AFM topographic scans of PIPOx backbone brushes via SI‐Cu^0^CRP (left), PIPOx‐*g*‐PMeOx BBBs after LCROP (middle) and swollen PIPOx‐*g*‐PMeOx BBBs in H_2_O (right), *Rms*=surface roughness. (b) Corresponding height profiles taken at scratches of the polymer layer. (c) Thickness column plot of POx brushes from (a). *S*_r_=swelling ratio, *σ*=grafting density.

The successful grafting of the PIPOx backbone brushes and further side‐chain extension with PMeOx are confirmed by water contact angle (*θ*) and Fourier‐transform infrared spectroscopy (FTIR). The FTIR spectra of PIPOx brushes show strong bands at 1660 and 1030 cm^−1^ assigned to the (C=N) and (C−O) stretching modes of oxazoline rings. After LCROP, the band around 1625 cm^−1^ refers to the (C=O) stretching mode of the amide function (amide I band) of PMeOx side chains. In addition, the band around 1425 cm^−1^ is assigned to CH_*x*_ deformation modes of backbones and side chains of POx BBBs (Figure S1). The water contact angle (*θ*) of α‐bromoisobutyryl bromide (BiBB) initiator‐functionalized SiO_2_ substrate is 69±2°. However, after first step SI‐Cu^0^CRP, the *θ* decreases to 53±2°, which is a typical value for PIPOx brushes as reported previously.5b After the second step LCROP, the *θ* changed slightly to 47±1° due to more hydrophilic PMeOx side chains. The dry thickness (*h*_dry_) of the PIPOx backbone brushes and PIPOx‐*g*‐PMeOx BBBs were measured by ellipsometry and AFM, respectively. As shown in Figure 2, the SI‐Cu^0^CRP at room temperature (RT) resulted in homogeneous PIPOx brushes with *h*_dry_=58±2 nm within 2 h. After 4 h LCROP, the *h*_dry_ of the resultant BBB increased to 110±4 nm. As reported previously, the highly crowded side chains lead to stretching of the bottle‐brush backbones and result in a layer height increase up to ≈100 %.5b The resultant PIPOx brush and POx BBB show homogeneous surface morphologies with low roughness (Rms) of 1.8 and 1.2 nm, respectively, as investigated by AFM (Figure S2 b and S2 f). The swollen thickness (*h*_swollen_=155±6 nm) of the BBBs in water was determined by liquid AFM. As such, the BBB grafting density, as estimated through the swelling ratio (*S*_r_ (%)=100 (*h*_swollen_−*h*_dry_)/*h*_dry_), is calculated to 0.42 chains nm^−2^ (Figure 2 b and Table S1).7h, 9 In comparison, only 15±1 nm PIPOx layer was obtained in 24 h SIPGP. After LCROP, the resultant PIPOx‐*g*‐PMeOx BBBs shows only 23±2 nm in thickness and 0.13 chains nm^−2^ in grafting density (Figure S3). Therefore, the SI‐Cu^0^CRP approach enables POx BBBs with significantly higher growth rate, thickness and grafting density than classical SIPGP approach.

### Grafting kinetics

**Figure 3:**
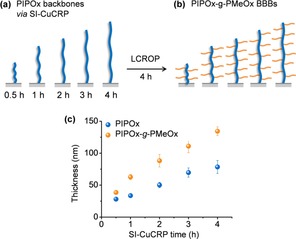
(a) Schematic of thickness variations of PIPOx backbone brushes as a function of SI‐Cu^0^CRP reaction time. (b) Resultant POx BBBs from (a). (c) Thickness plots of time‐thickness dependency of PIPOx backbones via SI‐Cu^0^CRP (blue) and resultant PIPOx‐*g*‐PMeOx BBBs after LCROP (orange).

The thickness variations of PIPOx backbone brushes as a function of SI‐Cu^0^CRP reaction time was further studied. As shown in Figure 3 a, PIPOx backbone brushes were synthesized through SI‐Cu^0^CRP and samples were taken out at different reaction times (i.e., 0.5, 1, 2, 3, and 4 h) and characterized by ellipsometry. As a result, the thickness of PIPOx brushes were 28±1, 34±1, 50±4, 69±7, 78±10 nm, respectively (Figure 3 a, Table S2). The PIPOx brush thickness reached to 50±4 nm in 2 h SI‐Cu^0^CRP. With longer reaction time, the growth rate decreased due to the reduced monomer concentration within the confined reaction set‐up. After second step LCROP, the dry thickness of resulted BBBs increased to 39±2, 63±4, 88±9, 111±10, 134±7 nm, respectively. (Figure 3 b, Table S2).

### Structured POx BBB surfaces

**Figure 4:**
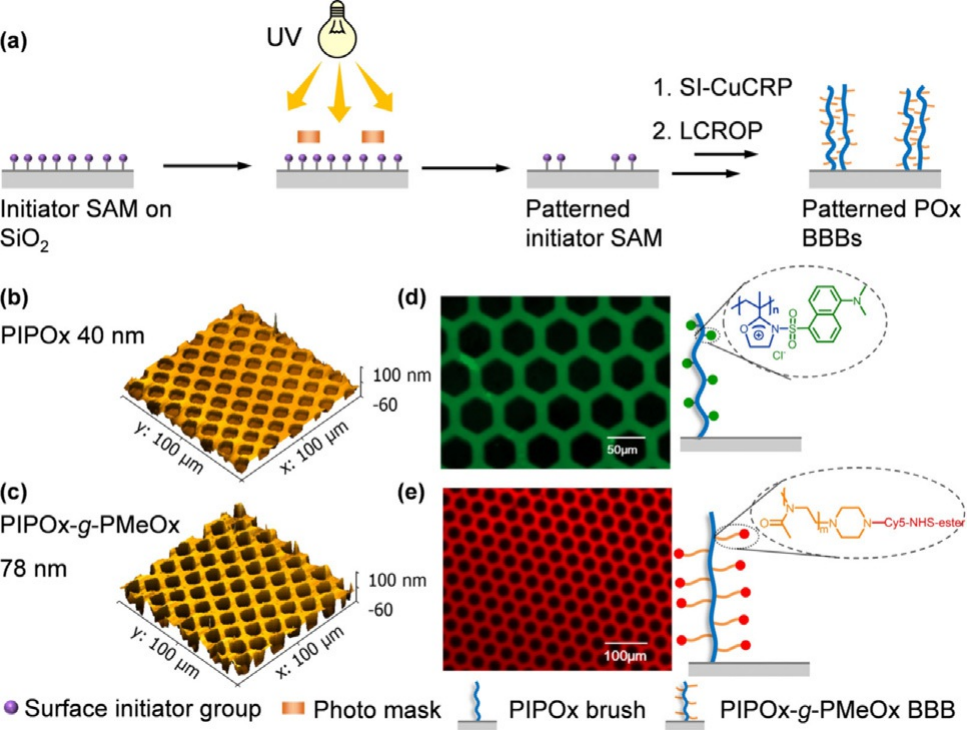
(a) Schematic illustration of the preparation of patterned polymer brushes. Structures can be introduced by UV light through a photo mask by removing uncovered initiator‐SAMs. (b) 3D AFM topographic scan of patterned PIPOx backbone brushes after 1.5 h SI‐Cu^0^CRP; (c) 3D AFM topographic scan of patterned PIPOx‐*g*‐PMeOx BBBs after 4 h LCROP. (d) Schematic illustration of coupling of the dansyl chloride with the PIPOx backbones (right) and epifluorescence microscopy image (left) (*λ*_ex_=440–470 nm). (e) Schematic illustration of coupling of the Cy5‐NHS‐ester with the PMeOx side chains (right) and epifluorescence microscopy image (left) (*λ*_ex_=525–550 nm).

Patterned POx BBB surfaces are of great interest because they can be used to spatially controlling protein adsorption, cell adhesion and molecular sensing.^10^ In the case of SI‐Cu^0^CRP approach, since the polymerization employs a self‐assembled monolayer (SAM) of surface‐anchored initiators, the patterned polymer brushes are simply accessible with patterned initiator‐SAMs, which are prepared by UV irradiation through a photomask (Figure 4 a).^11^ As shown in Figure 4 b, the PIPOx backbone brushes were selectively formed on initiator‐covered areas in SI‐Cu^0^CRP and then lead to patterned PIPOx‐*g*‐PMeOx BBBs after second step LCROP (Figure 4 c).

One of the most important characteristics of POx BBBs are the dual functionalities from backbones and side chains. In order to study the accessibility to such dual‐functionalities, two fluorescencent dyes were used to label the PIPOx backbones and the PMeOx side chains, respectively. Specifically, we employed dansyl chloride (maximal *λ*_em_ ≈525 nm)^12^ to label the PIPOx backbones, which can react with secondary amine groups and form stable sulfonamide. As shown in Figure 4 c, the fluorescent emission of PIPOx‐grafted areas presented a selective and fully functionalization of the PIPOx brushes. In order to prove the dansyl chloride is not physically adsorbed, the active group of this compound was protected by *N*‐*t*Boc and then reacted with the PIPOx patterns. As expected, no fluorescence emission was observed via epifluorescence microscopy (Figure S5). Afterwards, the PMeOx side chains were labelled by Cy5‐NHS‐ester (maximal *λ*_em_≈670 nm), which is a reactive dye for the labeling of amino‐groups.^13^ Thus, the Boc end group on side chains had to be deprotected using trifluoroacetic acid and then the deprotected BBBs were allowed to react with excess Cy5‐NHS‐ester in dry dimethylformamide with trimethylamine as a base for 24 h. After extensive cleaning to remove the excessive non‐reacted dye, the sample was investigated using epifluorescence microscopy, as shown in Figure 4 b, the fluorescent emission of labelled BBB side chains was presented. Therefore, the respective labelling of the backbones and side chains of POx BBBs with fluorescencent dyes demonstrate the dual functionalities of the POx BBB surfaces.

### POx BBB gradients

**Figure 5:**
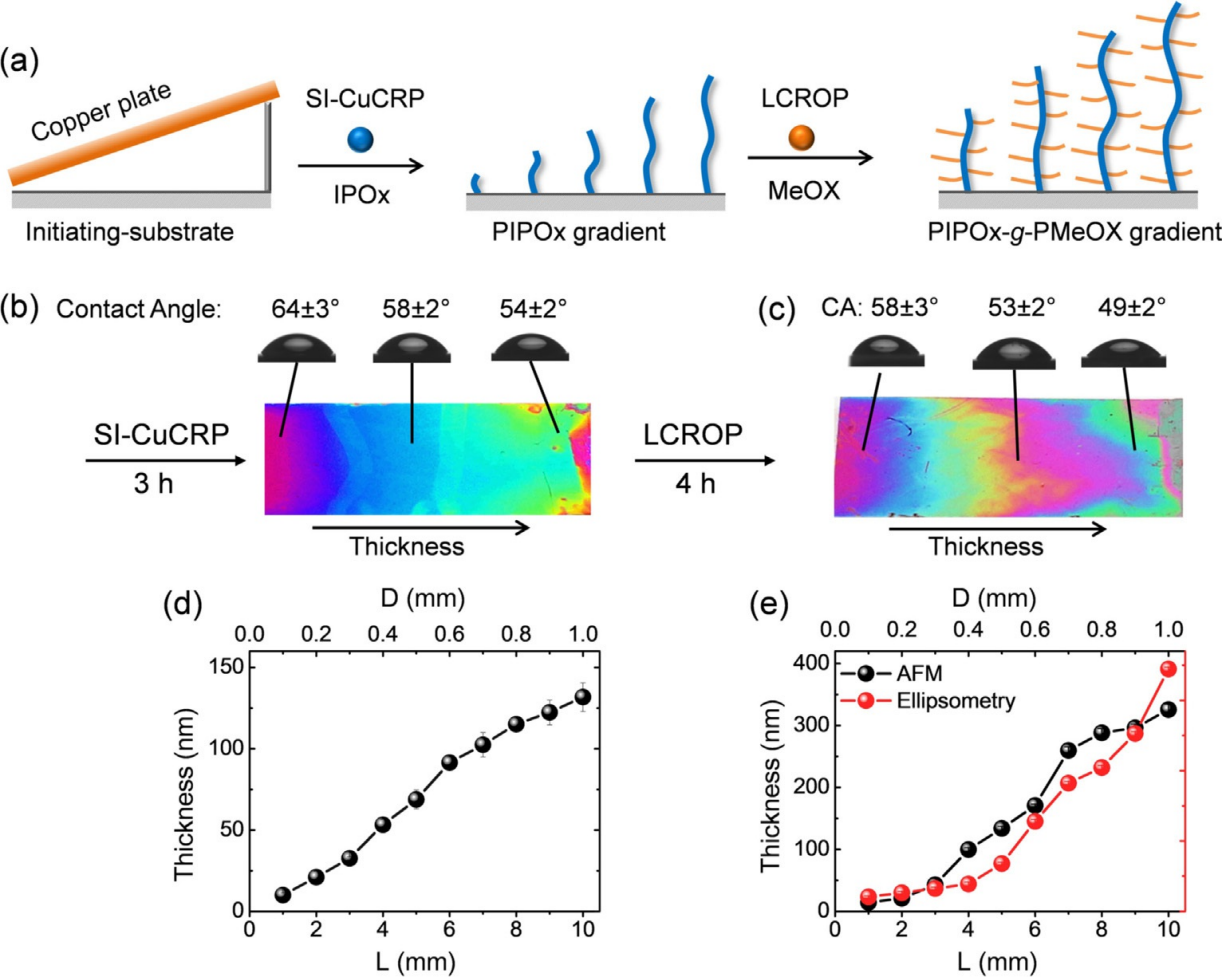
POx BBB gradient via two‐step approach: (a) Schematic illustration of the synthesis of PIPOx‐*g*‐PMeOx BBB gradient via SI‐Cu^0^CRP and LCROP. (b) Optical image and water contact angle data of resultant PIPOx gradient. (c) Optical image and water contact angle data of resultant POx BBB gradient. (d) Thickness plots of PIPOx backbone gradient as measured by ellipsometry. (e) Thickness plots of the POx BBB gradient as measured by AFM and ellipsometry, respectively.

Gradient polymer brushes are interesting anisotropic platforms to control the chemical, physical or morphological properties gradually across the surface.^14^ In conventional surface‐initiated polymerization (SIP) techniques, polymer brush gradients can be prepared by controlling polymerization time or initiator densities gradually along the surface.^15^ However, both strategies require tedious reaction steps and/or instruments, and consume a large amount of monomers. In the case of SI‐Cu^0^CRP, PIPOx brush gradients can be prepared straightforward using a tilted Cu‐plate (Figure 5 a). Because the gradual variation of the distance (D) between the Cu source and initiating‐substrate allows gradually changed polymerization rate and thus leads to polymer brush gradients.7c Even after side chain grafting, the gradient conformation was retained, but their thickness were systematically increased. For example, SI‐Cu^0^CRP with 3 h results in PIPOx brushes with gradient thickness ranging from 9 to 130 nm (Figure 5 d). The consecutive LCROP for 4 h enhances the gradient range to 14–320 nm as measured by AFM and ellipsometry (Figure 5 e and Table S4). It is worth to note that the polymer layers at edge are thicker (with 11–28 %) than that of middle positions (Figure S7). This is mainly due to the diffusion of monomer from outside to the reaction “chamber” (between copper plate and initiator substrate), which leaded to gradient concentrations of monomer along Y‐direction from edge to middle, and thus resulted in varied polymerization rate and brush thickness. The gradient brush thickness also results in gradual wetting properties as revealed by water contact angle measurements (Figure S6 b). Furthermore, we show that the POx BBBs can also be prepared through a one‐step SI‐Cu^0^CRP of beforehand prepared POx macromonomer. The methacrylic acid (MAA) terminated P(MeOx)_7_ macromomers (*M*_n_=536 g mol^−1^, PDI=1.10) were synthesized according to Kobayashi et al.^16^ (Figure S8) and characterized by gel permeation chromatography (GPC) and proton nuclear magnetic resonance (^1^H NMR).^16^ After 4 h SI‐Cu^0^CRP with tilted copper plate, the yield brush shows gradient thickness ranging from 6 to 60 nm (Table S4 and Figure S8). The grafting density of the resultant POx BBB was determined by liquid AFM as 0.19 chains nm^−2^ (Figure S9). Comparing to the two‐step synthesis described above, the brush thickness and grafting density of the POx BBB from one‐step approach are much lower due to the steric repulsion among macromonomer chains.^17^

## Conclusion

In this work, we report two approaches for the synthesis of well‐defined POx BBB surfaces via SI‐Cu^0^CRP. The two‐step synthesis consists of successive SI‐Cu^0^CRP and LCROP polymerizations that enables the fabrication of POx BBBs with high layer thickness (up to ≈300 nm) and grafting density (*σ*=0.42 chains nm^−2^). The synthesis of PIPOx backbones via SI‐Cu^0^CRP is more controllable and consumes minimum monomers in comparison to conventional SIPGP approach. The characteristic dual‐functionalities of resultant PIPOx‐*g*‐PMeOx BBBs are demonstrated by respective labelling of backbone and side chain with fluorescence dyes. In addition, the SI‐Cu^0^CRP also enables a one‐step approach to prepare well‐defined POx BBBs with PMeOx macromonomers, since the macromonomer (i.e. side chain) can be fully characterized before grafting polymerization. Regarding to the facile fabrication procedures (especially for patterns and gradients), bio‐ and dual‐functionalities, the POx‐based BBBs surfaces presented in this work are promising for various biomedical applications, for example, selectively tuning the surface adhesion, protein adsorption and cell behaviors.

## Conflict of interest

The authors declare no conflict of interest.

## Supporting information

As a service to our authors and readers, this journal provides supporting information supplied by the authors. Such materials are peer reviewed and may be re‐organized for online delivery, but are not copy‐edited or typeset. Technical support issues arising from supporting information (other than missing files) should be addressed to the authors.

Supplementary
