# Supplementary material for: Facile Fabrication of Bio‐ and Dual‐Functional Poly(2‐oxazoline) Bottle‐Brush Brush Surfaces
Source: Chemistry. 2020 Feb 12;26(12):2749–53. doi: 10.1002/chem.201905326 (PMC7064997; doi:10.1002/chem.201905326)
Supplement: Supplementary file 1 — Supplementary [file CHEM-26-2749-s001.pdf]

CHEMISTRY
A European Journal

Supporting Information

Facile Fabrication of Bio- and Dual-Functional Poly(2-oxazoline) Bottle-Brush Brush Surfaces

Yunhao Du,[a] Tao Zhang,*[b] Dan Gieseler,[a] Maximilian Schneider,[a] Daniel Hafner,[a] Wenbo Sheng,[a] Wei Li,[a] Fred Lange,[a] Erik Wegener,[a] Ihsan Amin,[c] and Rainer Jordan*[a]

chem_201905326_sm_miscellaneous_information.pdf

1. Materials and chemicals

The IPOx monomers were prepared according to Seeliger and co-workers.[1] The P(MeOx)$_7$-MAA macromonomers were prepared beforehand according to Kobayashi *et al.*.[2] Methyl triflate (MeOTf) and terminating reagents N-tert-butoxycarbonyl piperazine (N-Boc-piperazine) for living cationic ring opening polymerization (LCROP) were distilled prior to use. 2-Methyl-2-oxazolin was destabilized and dried *via* condensation over CaH_2 before use. Monomers and N-Boc-piperazine used for the LCROP and dry solvents were stored in a UNIlab glovebox from MBraun. Cu wafer (Siegert Wafer GmbH, Germany, 4 inch, 200 nm Cu layer, purity > 99.9 %, RMS < 10 nm) was consecutively washed with portions of ultrapure water, ethanol and DMSO under ultrasonication (5 min). The cleaned Cu plate was immediately used for Surface-Initiated Cu(0) Mediated Controlled Radical Polymerization (SI-CuCRP) experiments.[3] Silicon wafer pieces with a ~ 300 nm oxide layer were bought from MicroChemicals GmbH (Ulm, Germany). Methanol (Sigma-Aldrich, ≥99.8 %), ethanol (VWR Chemicals, absolute), dichloromethane (DCM, dry, Acros, ≥99.8 %), acetone (dry, Merck KGaA, ≥99.8 %), dimethylacetamid (DMAc, Sigma-Aldrich, for HPLC, ≥99.9 %), chloroform (Sigma-Aldrich, ≥99.8%), triethylamine (TEA, Sigma-Aldrich, ≥ 99.5 %), trifluoroacetic acid (TFA, Sigma-Aldrich, 99 %), 2-bromoisobutyryl bromide (BIBB, Sigma-Aldrich, 98 %), 3-amino-propyltriethoxysilane (APTES, Sigma-Aldrich, 99 %), 1,1,4,7,7-pentamethyl-diethylenetriamine (PMDETA, Acros, ≥98 %), dansyl chlorid (Sigma-Aldrich, ≥99 %), Cy5-NHS ester (Interchim) were used as received. All water used in the experiments was Millipore Milli-Q grade.

2. Instruments and characterizations

Atomic force microscopy (AFM): The topography and dry thickness of the polymer brushes was determined by atomic force microscopy in tapping mode (semi contact mode) on a customized NTEGRA Aura/Spectra from NT-MDT (Moscow, Russia) with a SMENA head. The used probes have a typical curvature radius of 6 nm, a resonant frequency of 47−150 kHz, and a force constant of 0.35−6.10 N/m. Editing, height determination and calculation of the surface roughness was done with the software Nova Px 3.2.5 from NT-MDT and Gwyddion for MS Windows from Czech Metrology Institute.

Elliposometry: The measurement were performed with a SE800 ellipsometer from SENTECH Instruments GmbH, equipped with a He–Ne laser source (λ = 632.8 nm) and a fixed angle of incidence of 60° at ambient conditions. The accumulated spectra were modeled using SpectraRay 3 software.

Water Contact Angle (WCA): The water contact angle measurements were performed with the Drop Shape Analysis System (DSA) 10 from Krüss GmbH to characterize the wettability of the polymer brushes. For each sample, individual measurements at three different spots were performed and averaged. WCA measurements were performed at room temperature with Milli-Q water. The contact angles were obtained using the tangent method fitting.

Gel permeation chromatography (GPC): GPC measurements were performed on a PL-GPC-120 (Polymer Laboratories) running under WinGPC software (PSS, Mainz, Germany) with two consecutive Gram columns (2 x 100 Å) using N,N-dimethylacetaminde (DMAc) (5 g/L LiBr, 70 °C, 1 mL/min) as eluent and calibrated against PMMA standards from PSS, Mainz, Germany.

Proton nuclear magnetic resonance (^{1}H NMR): ^{1}H NMR measurements were performed with a 500 MHz-device from Bruker. About 5 mg of each sample was weighed into a NMR tube and dissolved in 1 ml of deuterated solvent (Chloroform-d1 or D_2O).

Fourier transform infrared spectroscopy (FTIR): The FTIR characteristic spectra test was performed on a Bruker Tenrsor 27 FT-IR (Bruker, Billerica, USA) with a room temperature DTGS (deuterated triglycine sulfate) detector, mid-IR source (4000 to 400 cm^{-1}), and a KBr beamsplitter. Maximum resolution is 1 cm^{-1}. The adsorption spectra was analyzed with the software OPUS (Optical User Software) 7.5

Fluorescent microscopy: All the fluorescence images were taken with an epifluorescence microscope (Zeiss Axio Observer Z1m, Zeiss, Oberkochen, Germany). The fluorescence pictures of labeled patterned PIPOx brushes and PIPOx-*g*-PMeOx BBBs were taken at excitation *λ*: 440-470 nm (green) and 525-550 nm (red), respectively.

3. Experiment details

Self-assembled monolayer (SAM) of APTES-BiBB initiator: Silicon substrates were cleaned with oxygen plasma cleaner for 15 min and washed extensively with distilled water, and dried by dry nitrogen flow. The freshly cleaned substrates were amine-functionalized by immersed into a 5 % (v/v) aminopropyltrimethoxysilane (APTES) solution in dry acetone and ultrasonicated for 45 min under argon. After the SAM formation, the substrates were rinsed with acetone and dried by dry nitrogen flow. The substrate was then immersed in dry DCM (10 mL) under argon atmosphere. Afterwards, 0.2 mL triethylamine (TEA) was added before dropwise addition of 0.2 mL of 2-bromoisobutyryl bromide (BiBB) at 0 °C under argon. The mixture was then stirred under room temperature (RT) overnight. After the reaction the substrate was rinsed with DCM, water, ethanol, and acetone, and then dried with a dry nitrogen flow. The immobilization of the initiator SAM was finished for subsequent SI-CuCRP. Water contact angle after SAM immobilization: θ = 69 ± 2 °. SAM thickness: ~ 2 nm measured by ellipsometry.

Synthesis of 2-*iso*-propenyl-2-oxazoline (IPOx) monomer: The IPOx monomer was prepared according to Seeliger and co-workers.[1] 10.0 g formaldehyde (HCHO, 0.33 mol, 1.1 eq.) and 29.73 g 2-ethyl-2-oxazoline (EtOx, 0.3 mol, 1 eq.) was mixed in a 500 mL round-bottomed flask with reflux-condenser. After addition of 0.759 g triethylamine (TEA, 75 mmol), the mixture was heated to 50 °C under stirring for 20 min. The mixture was then heated to 120 °C and stirred for 3 h under nitrogen atmosphere. The middle product hydox was distilled at 90 °C under reducing pressure 0.04 mbar. The obtained hydrox was then mixed with 3.0 g NaOH (75 mmol). After addition of few amount hydrochinon, the mixture was then heated to 120 °C and stirred for 4 h under nitrogen. The IPOx product was distilled at 120 mbar and 80 °C, which is colorless liquid. **Yield:** 12.1 g (36 %)

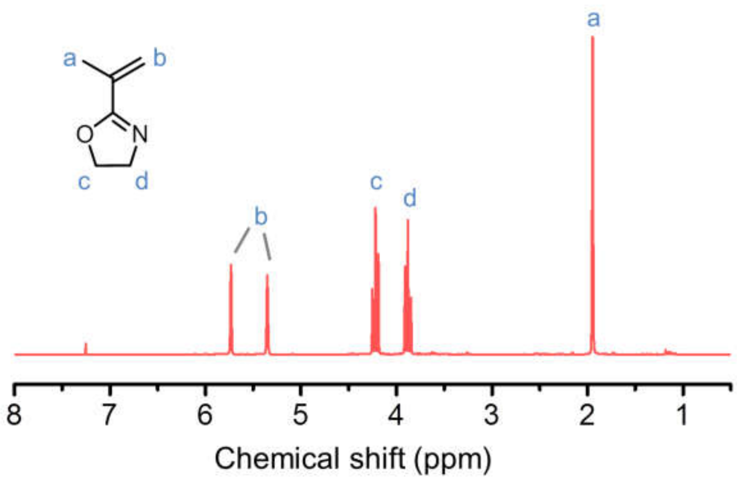

^{1}H NMR ($CDCl_3$, 500MHz): *δ* (ppm) = 1.96 (t, 3H, CH_3-C=CH_2-), 3.93 (t, 2H, =N-CH_2-CH_2), 4.27 (t, 2H, -CH_2-CH_2-O-), 5.42 (s, 1 H, -C=CH_2), 5.79 (s, 1 H, -C=CH_2).

Synthesis of P(MeOx)$_7$-MAA macromonomer: The P(MeOx)$_7$-MAA macromonomer was prepared according to Kobayashi *et al.*[2] 3.7926 g (23.1 mmol, 1 eq.) of MeOTf were weighed into a Schlenck-tube and dissolved in 30 mL ACN under argon atmosphere in glovebox. Then 10.6556 g (125.2 mmol, 5.4 eq.) of MeOx were added. The mixture was discharged from the glovebox and heated in an oil bath at 90 °C for 30 minutes. Afterwards the reaction was allowed to cool down to room temperature. 4.9776 g (57.8 mmol, 2.5 eq.) of MAA and 5.84 g (57.7 mmol, 2.5 eq.) of Et_3N were added under argon protection. The mixture was heated up to 70 °C and stirred overnight. Then, 6 g of K_2CO_3 were added and the reaction was stirred at room temperature overnight. The solution was decanted and filtrated. ACN was removed under vacuum and the remaining solid was dissolved in 20-25 mL methanol. The product

was then precipitated into 750 mL diethyl ether. The solid was separated from the solvent and dried under vacuum. The product was purified by 2 more time precipitating and the solid was dried in freeze dryer to obtain the product as light sticky yellow powder. **Yield:** 9.19 g (70 %).

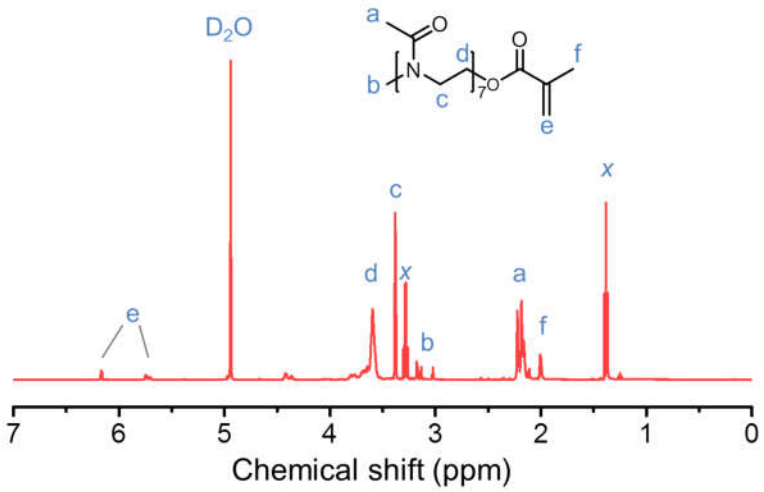

^{1}H NMR (D_2O, 500 MHz): *δ* (ppm) = 1.95 (m, 3 H, CH_3-C=CH_2), 2.21 (br, 21 H, CH_3-CO-), 3.03 (d) and 3.19 (dd, 3 H, CH_3-N-), 3.42 (br, 26 H, -N-CH_2-CH_2-), 3.70 (br, 2 H, -CH_2-CH_2-O-), 5.69 (m) and 6.18 (s, 2 H, -C=CH_2).
$\boldsymbol{M_{theo}}$ = 525 g/mol, $\boldsymbol{M_n}$ ***(GPC)*** = 536 g/mol, ***PDI*** = 1.10.

SI-CuCRP of PIPOx on silicon oxide: The polymer brushes were prepared by a planar silicon wafer piece modified with a self-assembled monolayer (SAM) of a typical ATRP-initiator (surface-anchored 2-bromoisobutyryl bromide, APTES-BiBB) were sandwiched with a copper plate and immerged into pre-degassed reaction solution of monomer (0.5 mL IPOx, 4.7 mmol), solvent (millipore-Q water 1 mL, methanol 0.5 mL), and ligand PMDETA (20 µL, 0.096 mmol) in a test tube. After certain hour's polymerization at RT, the samples were taken out of the test tube and rinsed with water and ethanol and then dried with nitrogen flow for further characterizations.

SIPGP of PIPOx on silicon oxide: The silicon oxide substrate was cleaned with plasma cleaner for 15 min. Then the substrate was rinsed with Millipore-Q water and submerged into the solution of dry acetone and 5% (v/v) APTES. After 45 min ultrasonification the substrate was rinsed with acetone, ethanol and Millipore-Q water thoroughly. In a test tube put in 1 mL IPOx and degassed with nitrogen for 30 min to remove the oxygen, the cleaned substrate was submerged into the monomer and put under UV lamp (wavelength ~ 350 nm). The SIPGP was carried out under nitrogen for certain time. After the SIPGP, the substrate was taken out and rinsed with water and ethanol thoroughly.

Surfaced initiated LCROP to synthesis PIPOx-*g*-PMeOx BBBs: In glove box an initiating-substrate was submerged in a solution of 2 mL ACN with an excess amount of MeOTf (30 mg) at approximately -35 °C under a dry argon atmosphere. After stirring for 5 h at 0 °C, the mixture was allowed to equilibrate to RT and stirred overnight. 1 g monomer MeOx (0.01mol) was added to the reaction flask afterwards. The reaction solution was heated at 80 ◦C and stirred for 4 h. Then, the solution was cooled down to 0 °C, and 1 mL N-tert-butyloxycarbonyl- piperazine dissolved in ACN solution (200 mg/mL) was added under argon atmosphere and the solution was stirred overnight at RT. Afterwards, an excess amount of potassium carbonate was added to the solution and stirred overnight to neutralize excess cations. The substrate was removed from the reaction solution and cleaned by ultrasonications in Milli-Q water, ethanol, and ethyl acetate for 1 min each.[4] The thickness data are summarized in Table S1. In order to estimate the grafting density of the BBBs, we employed AFM in liquid media to investigate their swollen thickness. The polymerization degree N and grafting density of the BBBs can be calculated according to equation (2) and (3).[5] The swollen ratio and grafting density are shown in **Table S2**.

In order to estimate the side chain length as well as molecular weight. The reaction solution after LCROP was purified and precipitated, the obtained polymers were tested by GPC using DMAc as eluent. The data are shown in Figure S4, the change of the ratio between side chain monomer and initiator does not influence the polymer brush heights on surface obviously. The measured molecular weights of the polymers in solution are in good agreement with the theoretical molar mass. The dispersity of the polymer varies from 1.1 to 1.3 (**Table S3**).

Deprotection of the Boc group: The silicon substrate with PIPOx-*g*-PMeOx-Boc BBB was submerged in a solution of 1 mL trifluoroacetic acid (TFA) and 1 mL chloroform. The mixture was stirred at RT for 3 h. Then, the substrate was neutralized in a 5 % $NaHCO_3$ water solution for 2 h. Finally, the substrate was washed by ultrasonification in water, ethanol and ethyl acetate for 1 min each.[4b]

SI-CuCRP for the synthesis of polymer brush gradients: A silicon wafer piece modified with an APTES-BiBB-SAM was sandwiched with a copper plate with one side in direct contact and the other spaced at a distance D = 1 mm using a 1 mm thick stripe of glass (sample length L = 10 mm). The setup was fixed with clamps and emerged into respective reaction solutions (*eg.* **Figure 5a**). The thickness data and surface wettability are summarized in **Table S3**.

Patterned APTES-BiBB Initiator-SAM: The patterned initiator was prepared via UV irradiation (200 W Hg (Xe) lamps, LOT-oriel, Germany) through a transmission electron microscopy (TEM) grid as photomask.[6] The samples were clamped with Cu TEM grids with various structures (Plano, Germany) and irradiated for 45 min at a distance of ~ 10 cm. Through this method the initiator group on the substrate was removed in uncovered regions and the polymer brushes were grafted selectively to generate patterned structures. After SI-CuCRP and LCROP, the patterned structures and polymer thickness were determined by AFM. The thickness of the patterned PIPOx brush reached to 40 nm after 1.5 h SI-CuCRP. After 4 h LCROP, the thickness reached up to 78 nm.

Fluorescence labeling of the PIPOx brushes: 27 mg dansyl chloride (0.1 mmol) and 50 µL TEA as base was added in 2 mL dry dimethylformamide (DMF) under argon atmosphere and stirred for 10 min to form a light green solution. The substrate with PIPOx backbone brushes was submerged in the solution under argon and stirred for 24 h in dark at RT. The substrate was then taken out and washed by ultrasonification in DMF, water and ethanol for 1 min each. In order to prove that the dansyl chloride is not physically adsorbed, but chemically coupled, the active group of the dansyl chloride was protected by N-tert-Boc and then reacted with the PIPOx patterns. As expected no fluorescence emission was observed under microscopy, which further confirmed the successful coupling of the dansyl chloride with PIPOx brushes.

Fluorescence labeling of the PIPOx-*g*-PMeOx BBBs: ca. 1 mg Cy5-NHS-ester was dissolved in 2 mL dry DMF with 50 µL TEA as base under argon to form dark blue solution, the substrate with PIPOx-*g*-PMeOx BBBs was submerged into the solution and stirred in dark at RT for 24 h. The sample was then taken out and washed by ultrasonification in DMF, water and ethanol for 1 min each.

4. Supporting results

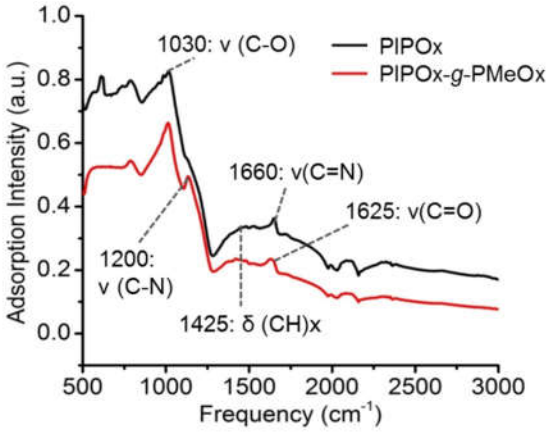

Figure S1: FTIR spectra of PIPOx back bone brush and PIPOx-*g*-PMeOx BBB.

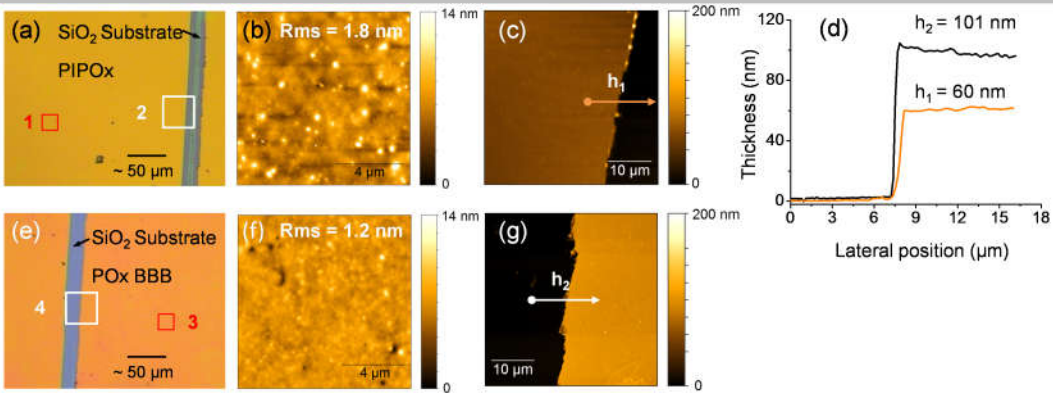

Figure S2 | Topological AFM scans of POx BBB surface. (a) Optical images of obtained PIPOx brush surface. **(b)** Detailed topological AFM scan (10 x 10 µm^2) of POx BBB on spot 1 as indicated in (a) by red square, *Rms* = 1.8 nm. **(c)** Topological AFM scan on spot 2 as indicated in (a) by white square. **(d)** Height profiles taken at scratches of the polymer surface at spot 2 and 4. **(e)** Optical images of obtained POx BBB surface. **(f)** Detailed AFM scan (10 × 10 µm^2) at spot 3 indicated in (e) by red square, the roughness *Rms* = 1.2 nm. **(g)** Topological AFM scan of POx BBB on spot 4 as indicated in (e) by white square.

In order to further prove that the POx brushes are morphologically homogeneous, we performed atomic force microscopy (AFM) topological scans on different positions of the PIPOx brush and POx BBB surfaces (Figure S2). The obtained PIPOx and POx BBB surfaces are quite homogeneous at microscopic scale as revealed by optical microscopy images (Figure R1a and R1e). The PIPOx surface shows roughness of 1.8 nm with 60 nm thickness (Figure S2b) and the 101 nm-thick POx BBB surface shows roughness of 1.2 nm (Figure S2f)

Table S1: Thickness and contact angle data of PIPOx back bone brushes and POx BBBs *via* classical two-step synthesis (SIPGP + LCROP) and new two-step synthesis (SI-CuCRP + LCROP).

Sample	React-Time	PIPOx	θ (°)	h_{dry} (nm)	LCROP Time	PIPOx-g-PMeOX	θ (°)	h_{dry} (nm)	$h_{swollen}$ (nm)	σ (chains /nm^2)
SIPGP	24 h	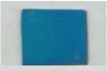	49 ± 1	15 ± 1	4 h	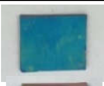	45 ± 3	23 ± 2	66 ± 3	0.13
SI-CuCRP	2 h	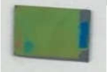	53 ± 2	58 ± 2		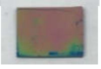	47 ± 1	110 ± 3	155 ± 6	0.42

θ: Water drop contact angle; σ: Grafting density of the polymer chains.

$h_{\text{swollen}} = (12/\pi)^{1/3} N \sigma^{1/3} (\omega/v)^{1/3}$ (1)

$N = [1.074(h_{swollen})^{3/2}]/[(h_{dry}\ (\text{Å}^2))^{1/2}]$ (2)

$\sigma = \rho_0 h_{dry} \mathrm{N_A} / N\ \mathrm{M_0}$ (3)

Sr = Swollen Ratio; *N* = polymerization degree, according to equation (2), N_{SIPGP} = 1225, $N_{SI\text{-}CuCRP}$ = 1846; ρ_0=Density of the monomer (MeOx, 1.005 g/mol); excluded volume parameter (ω = 7 Å^3), *a* is constant, $v = (a^2/3)^{-1}$, where a represents the Kuhn length of the monomer unit. $\mathrm{N_A}$=Avogadro constant (~ 6.022×10^{23}). $\mathrm{M_0}$ is the molecular mass of MeOx = 85.10 g/mol. The constant related to the excluded volume parameter, was always set as 1.074 independent of the type of monomers.[5]

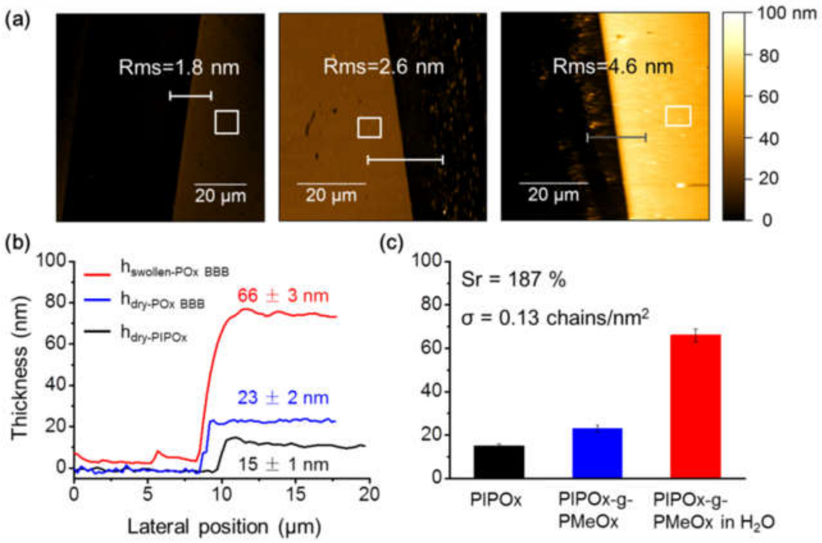

Figure S3. **(a)** AFM topographic scans of PIPOx back bone brushes via SIPGP (left), *Rms* = surface roughness, PIPOx-*g*-PMeOx BBBs after LCROP (middle) and swollen PIPOx-*g*-PMeOx BBBs in H_2O (right). **(b)** Corresponding height profiles taken at scratches of the polymer layer. **(c)** Thickness plots of POx polymer layers from (b). *Sr* = swelling ratio, *σ* = grafting density.

Table S2. Thickness of PIPOx backbone brushes *via* SI-CuCRP for 0.5 ~ 4 h and resultant PIPOx-*g*-PMeOx-Boc BBBs.

SI-CuCRP time	PIPOx Layer Thickness (nm)	PIPOx-*g*-PMeOx-Boc Layer thickness (nm)	Increasing Ratio
0.5 h	28 ± 1	39 ± 2	39 %
1 h	34 ± 1	63 ± 4	85 %
2 h	50 ± 4	88 ± 9	76 %
3 h	69 ± 7	111 ± 10	61 %
4 h	78 ± 10	134 ± 7	72 %

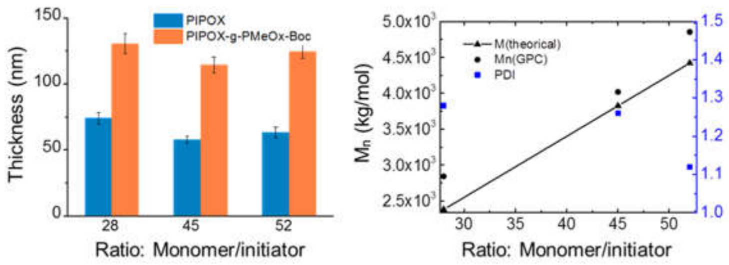

Figure S4: Left: Thickness data of PIPOx-*g*-PMeOx-Boc BBB applying different monomer/initiator ratio; Right: Molecular weight and polydispersity index (PDI) of the polymers in reaction solution measured by GPC.

Table S3: GPC data of the polymeric PMeOx side chains in reaction solution.

PIPOx thickness (nm)	Monomer/Initiator of LCROP	PIPOx-*g*-PMeOx Thickness (nm)	M_n theory (g/mol)	M_{nGPC} (g/mol)	*PDI*
74 ± 4	28	130 ± 7	2553	2842	1.28
58 ± 3	45	114 ± 6	3998	4019	1.26
65 ± 4	52	125 ± 5	4593	4855	1.13

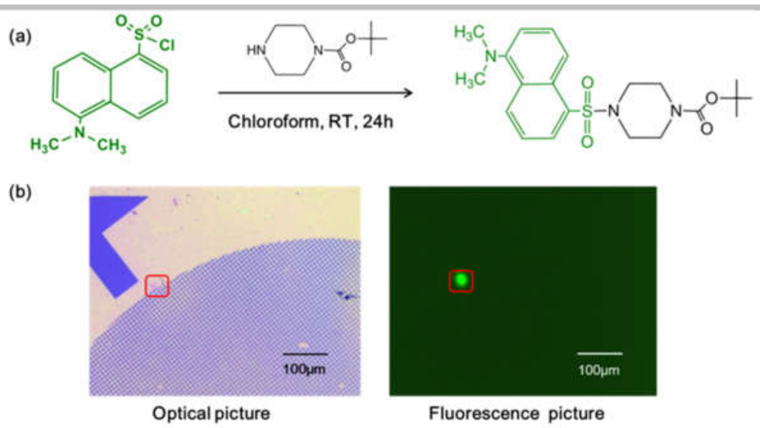

Figure S5: (a) Schematic illustration of the protection of the dansyl chloride. **(b)** Optical image (left) and fluorescence image of the sample taken by microscopy (Excitation λ: 335-383 nm).

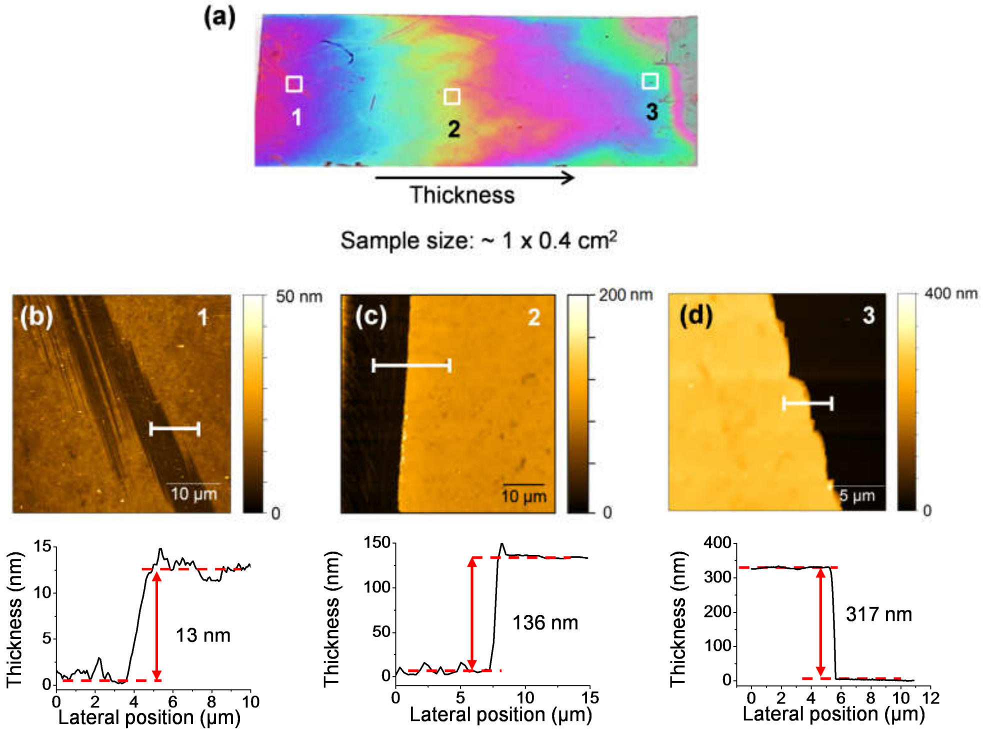

Figure S6 | Exemplar topological AFM scans along POx BBB gradient *via* two-step approach. (a) Optical images of the POx BBB gradient. **(b), (c) and (d)** Topographic AFM scans on 3 different spots along the POx BBB gradient as indicated in (a) by white squares.

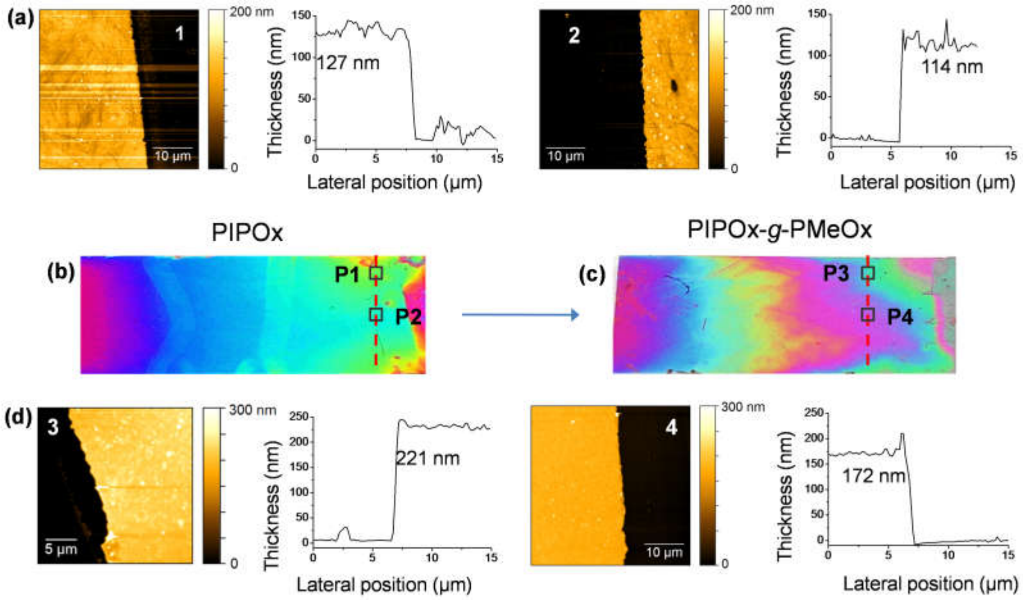

Figure S7 | Topological AFM scans along the Y-direction of the gradients. (a) Left: topological AFM scan and height profile of PIPOx gradient on spot 1 as indicated in the optical image in (b), right: topological AFM scan and height profile of PIPOx gradient on spot 2 as indicated in the optical image in (b). **(b)** Optical image of PIPOx gradient after SI-CuCRP. **(c)** Optical image of POx BBB gradient after LCROP. **(d)** Left: topological AFM scan and height profile of POx BBB gradient on spot 3 as indicated in the optical image in (c), right: topological AFM scan and height profile of POx BBB gradient on spot 4 as indicated in the optical image in (c).

We performed AFM scans on different positions along Y-direction of both PIPOx brush and POx BBB gradients (Figure S7). We found that the polymer layers at edge (P1 and P3) are 11 - 28% thicker than that of middle positions (P2 and P4). One possible reason is that the concentration of monomer along Y-direction is different due to lateral diffusion. The unique experimental set-up of SI-CuCRP (Figure 1 b) creates a confined polymerization/reaction "chamber" between the copper plate and initiating-substrate. Once the monomer in the confined "chamber" is consumed by polymerization, the outside monomers diffuse into the "chamber", which therefore creates gradient concentrations of monomer along Y-direction from edge to middle and results in varied polymerization rate and brush thickness.

Table S4. Thickness and wettability data of the POx BBB gradients *via* two-step and one-step synthesis using SI-CuCRP.

Polymer brush gradients	Lateral Position [a] (L) (mm)	Distance (D) (mm) [b]	Dry Thickness (nm) (AFM)	Contact Angle (*θ*) (°)
PIPOx-g-PMeOx *via* SI-CuCRP and LCROP	1	0.1	14 ± 2	58 ± 3
	2	0.2	21 ± 2	
	3	0.3	42 ± 3	
	4	0.4	99 ± 3	
	5	0.5	133 ± 4	
	6	0.6	171 ± 2	53 ± 2
	7	0.7	259 ± 6	
	8	0.8	288 ± 7	
	9	0.9	296 ± 6	
	10	1	321 ± 9	49 ± 2
$P(MeOx)_7$-MAA *via* SI-CuCRP	1	0.1	6 ± 1	24 ± 2
	2.8	0.28	14 ± 1	
	4.6	0.46	24 ± 3	
	6.4	0.64	39 ± 3	
	8.2	0.82	45 ± 5	
	10	1	55 ± 2	

a: The lateral scratches were inflicted onto the BBB gradient carefully by a plastic tweezer using a common ruler to measure the distance between adjacent scratches, the lateral position might not be so precise as described in the table. b: The distance (D) between copper plate and initiating-substrate was calculated according to the estimated lateral position.

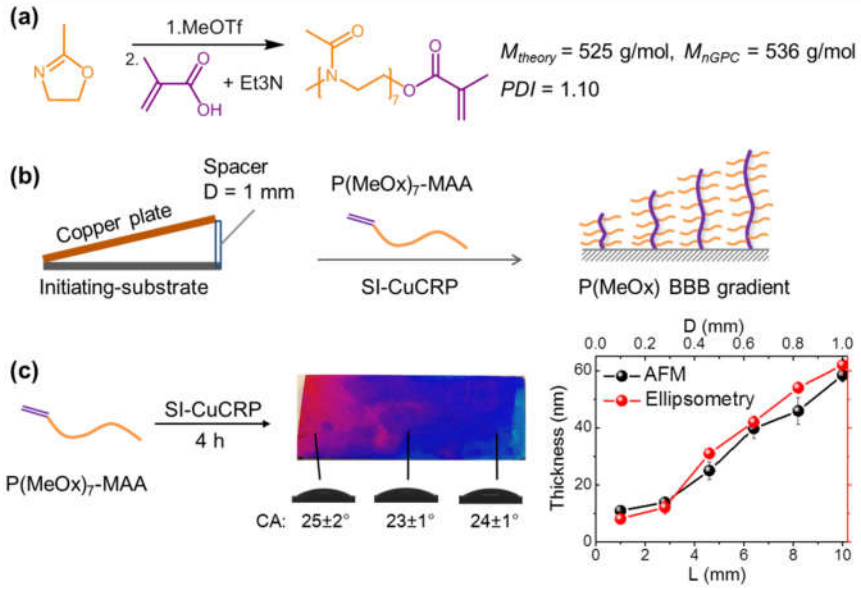

Figure S8: (a) Synthesis of P(MeOx)$_7$-MAA macromonomer and GPC data. **(b)** Schematic illustration of synthesis of POx BBB gradient *via* one-step SI-CuCRP. **(c)** Optical image of the resultant PIPOx-*g*-PMeOx BBB gradient and thickness plot as measured by AFM and ellipsometry, respectively.

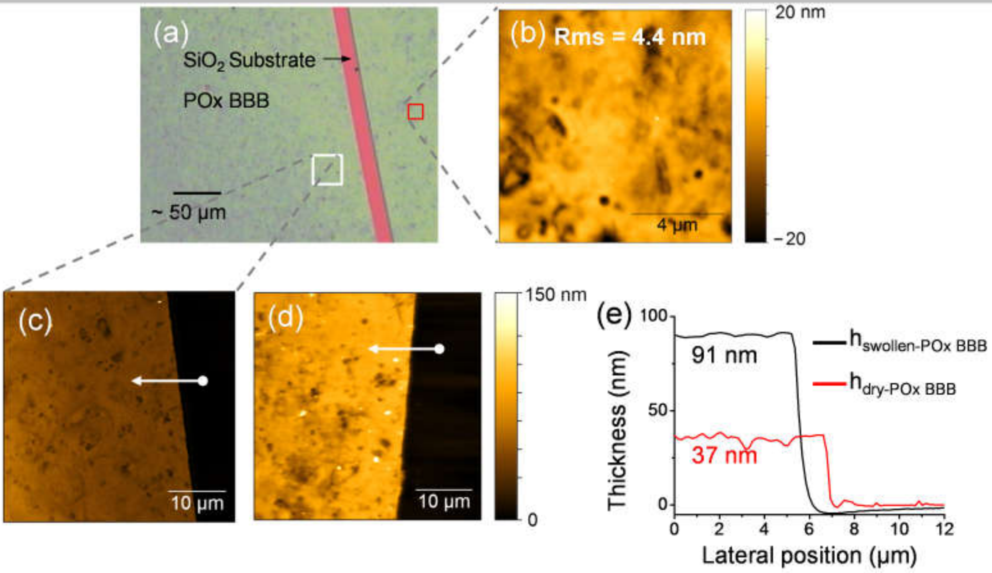

Figure S9 | Topological AFM scans of POx BBB surface from one-step approach. (a) Optical image of the POx BBB. **(b)** Detailed topological AFM scan (10 × 10 µm^2) on spot indicated in (a) by red square, the roughness *Rms* = 4.4 nm. **(c)** Topological AFM scan of POx BBB in dry state on the spot indicated in (a) by white square. **(d)** Topological AFM scan of POx BBB in H_2O. **(e)** Height profiles taken at scratches of the POx BBB surface, h_{dry} = 37 nm, $h_{swollen}$ = 91 nm.

To further study the POx BBB surface via one-step approach, AFM scans were employed to investigate the grafting density and surface morphologies. The grafting density calculated from swelling ratios (146%, Figure S9e) is ca. 0.19 chains/nm^2, which is considerably lower than that *via* two-step approach (0.42 chains/nm^2). This is because the monomer used in one-step approach is macromomer (M_n = 536 g/mol, *PDI* = 1.10), which is difficult to polymerize on surface due to larger steric hindrance and therefore leads to lower grafting density.

The POx BBB surface *via* one-step approach showed lower thickness (h_{dry} ~ 37 nm) and higher roughness (*Rms* = 4.4 nm) in comparison with that of two-step approach (h_{dry} ~ 100 nm, *Rms* = 1.2 nm). The water contact angel *(θ)* difference of two POx BBB surfaces are mainly attributed to the termination reagent used in the LCROP process in two-step approach (Figure 1). The N-tert-butoxycarbonyl piperazine (N-Boc-piperazine) has a hydrophobic Boc end-group, which contributes to the higher water contact angle (*θ* = 49 ~ 58°) of POx BBB from two-step approach. The POx BBB resulted from one-step approach has dual-functionalities as well, because the backbone brush and side chain of POx BBB can be functionalized, respectively.

References

[1] W. Seeliger, E. Aufderhaar, W. Diepers, R. Feinauer, R. Nehring, W. Thier, H. Hellmann, *Angew. Chem. Int. Ed.* **1966**, *5*, 875-888.

[2] S. Kobayashi, E. Masuda, S. Shoda, Y. Shimano, *Macromolecules* **1989**, *22*, 2878-2884.

[3] a) T. Zhang, Y. Du, F. Muller, I. Amin, R. Jordan, *Polym. Chem.* **2015**, *6*, 2726-2733; b) T. Zhang, Y. Du, J. Kalbakova; R. Schubel, R. D. Rodriguez, T. Chen, D. Zahn, and R. Jordan, *Polym. Chem.* **2015**.

[4] a) N. Zhang, T. Pompe, I. Amin, R. Luxenhofer, C. Werner, R. Jordan, *Macromol. Biosci.* **2012**, *12*, 926-936; b) N. Zhang, M. Steenackers, R. Luxenhofer, R. Jordan, *Macromolecules* **2009**, *42*, 5345-5351.

[5] a) Y. Che, T. Zhang, Y. Du, I. Amin, C. Marschelke, R. Jordan, *Angew. Chem. Int. Ed.* **2018**, *57*, 16380-16384; b) R. Jordan, A. Ulman, J. F. Kang, M. H. Rafailovich, J. Sokolov, *J. Am. Chem. Soc.* **1999**, *121*, 1016-1022.

[6] T. Chen, I. Amin, R. Jordan, *Chem. Soc. Rev.* **2012**, *41*, 3280-3296.
